# Account of a Living Female Child, Aged Two Months and Ten Days, Which Had Been Born without Limbs, and Which Was Lately Presented to the Society of the School of Medicine, at Paris

**Published:** 1803-01-01

**Authors:** 


*Account of a living Female Child, aged two Months and ten Days, which had been born without Limbs, and which was lately presented to the Society of the School of Medicine, at Paris.*

THE lower extremities of this subject were indicated by two small protuberances, situated in little cavities or impressions of the skin. Of the upper extremities, a very short arm was to be seen on the right side; but on the left, there was nothing but an appendix, half as short as the former. On the skin, which covered these two rudiments of arms, a scar or cicatrix of no very deep impression was observed. All the other parts of the trunk were well formed. The mother did not remember to have experienced any particular accident during pregnancy, nor could any traces of the separation of the limbs be discovered in the lochiæ. The child died three days after its having been examined by the society. Cit. Dupuytren, on dissecting it, observed, observed, that all the muscles terminated at a certain distance from the above rudiments; the humerus of the arm was entire, and ended in facets or surfaces of articulation. On the left side there was only that part of the os humeri which belongs to the scapula; it terminated in a sort of conus which was closely united with the cicatrix of the skin by means of a very strong cellular texture. In the appendices of the pelvis, which resembled warts, nothing but a cellular texture could be seen, though at the basis there was a bony portion, which was covered with strong tela cellulosa; in which, traces of the principal nerves and vessels could with difficulty be observed. The study of monstrosities, in which the laws of formation, generally adopted by nature, are so variously changed, and the numerous deviations from the established forms of nature, is not a little interesting for physiologists, and is indeed, something more than merely a matter of curiosity. It is from these aberrations of nature, that we may hope to penetrate into the mystery of those laws, which superintend the formation of animated beings; or at least we may, by a due attention to these anomalies, be enabled to guard ourselves against the erroneous hypotheses which certain physiological works contain on that mysterious subject.

